# A preoperative nomogram for sepsis in percutaneous nephrolithotomy treating solitary, unilateral and proximal ureteral stones

**DOI:** 10.7717/peerj.9435

**Published:** 2020-06-29

**Authors:** Yang Xun, Yuanyuan Yang, Xiao Yu, Cong Li, Junlin Lu, Shaogang Wang

**Affiliations:** Department of Urology, Tongji Hospital, Tongji Medical College, Huazhong University of Science and Technology, Wuhan, China

**Keywords:** Percutaneous nephrolithotomy, Infection, Nomogram, Urolithiasis, Albumin globulin ratio

## Abstract

**Background:**

Postoperative sepsis is a lethal complication for percutaneous nephrolithotomy (PCNL). An early predictive model combined local and systemic conditions is urgently needed to predict infectious events. We aim to determine the preoperative predictors of sepsis after PCNL in patients with unilateral, solitary, and proximal ureteral stones.

**Methods:**

A total of 745 patients who underwent PCNL between January 2012 and December 2018 were retrospectively enrolled. Sepsis was defined based on the International Sepsis Definitions in 2001, and the preoperative factors were compared between the non-sepsis and sepsis groups. Univariable analysis and multivariable logistic regression analysis were conducted to determine the predictors for sepsis after PCNL. A nomogram was generated using the predictors.

**Results:**

In this study, 35 patients (4.7%) developed sepsis after PCNL. Univariate analysis showed that post-PCNL sepsis was associated with the female, lower albumin, higher globulin, lower albumin globulin ratio (AGR < 1.5), preoperative fever, leukocytosis (WBC ≥ 10,000 cells/μL), positive urine culture, leukocyturia (≥50 cells/μL) and positive urine nitrite. Multivariate logistic regression analysis suggested that AGR < 1.5 (odds ratio [OR] = 5.068, 95% confidence interval [CI] [1.135–22.624], *P* = 0.033), positive urine culture (OR = 3.243, 95% CI [1.162–9.047], *P* = 0.025), leukocytosis (OR = 3.706, 95% CI [1.444–9.512], *P* = 0.006) and female (OR = 2.529, 95% CI [1.127–5.672], *P* = 0.024) were independent risk factors for sepsis. A nomogram was generated and displayed favorable fitting (Hosmer–Lemeshow test *P* = 0.797), discrimination (area under receiver operating characteristic curve was 0.807), and clinical usefulness by decision curve analysis.

**Conclusions:**

Patients with certain preoperative characteristics, such as female, lower AGR, positive urine culture, and leukocytosis, who undergo PCNL may have a higher risk of developing sepsis. A cautious preoperative evaluation and optimized treatment strategy should be considered in these patients to minimize infectious complications.

## Introduction

Urolithiasis is a common urologic disease with a prevalence rate of 5.8% in the general population, and the recurrence rate has been shown to reach 60% within 10 years (Zeng et al., 2017; Scales et al., 2012). The treatment options for stone disease range from open surgery to endoscopic surgery, such as percutaneous nephrolithotomy (PCNL) and ureteroscopy (URS) (Heinze, Gozen & Rassweiler, 2019). Generally, PCNL results in higher stone-free rate than URS, at the expense of elevated complication rate and prolonged hospital stays, without difference in secondary interventions (De et al., 2015; Chung et al., 2019). However, infection remains a main complication that can cause longer length of stay and even lethal sepsis shock (Kreydin & Eisner, 2013). Therefore, establishing a predictive model, especially in the early stages of hospitalization, is imperative to help develop prevention strategies.

The risk factors for sepsis after PCNL were systematically summarized (Kreydin & Eisner, 2013). The preoperative factors comprised the following: positive urine culture, female sex, nephrostomy, urinary diversion, stone size, hydronephrosis, diabetes and complicated stone. Among these, the local urological condition is considered to be a critical factor related to infectious complications. Additionally, new factors for infection have emerged in recent years. These factors, such as C-reactive protein, albumin and procalcitonin, show strong predictive value by reflecting the systematic condition of the patient (Xu & Guo, 2019; Rivera et al., 2016).

Currently, a preoperative model, integrating local and systematic conditions, is scarce to evaluate the probability of sepsis. The purpose of this study was to analyze post-PCNL sepsis in solitary, unilateral, and proximal ureteral stone patients. Furthermore, the results of this study were used to develop a preoperative risk factor nomogram that may help urologists identify patients who are more likely to develop sepsis.

## Materials and Methods

The study was conducted under the approval of the Ethics Committee of Tongji Medical College, Huazhong University of Science and Technology (2019S1035). We retrospectively enrolled 745 patients who underwent PCNL from January 2012 to December 2018. The inclusion criteria were the following: (1) PCNL was performed to treat unilateral, solitary, and proximal ureteral stones; and (2) age ≥18 years. Stone size was bigger than 10 mm. The exclusion criteria were anatomical renal abnormalities (horseshoe kidney, solitary kidney, transplant kidney and kidney duplication). Patients imaging, including abdominal computed tomography CT, confirmed the presence and size of proximal ureteral stone (above the fourth lumbar spine or in the ureteropelvic junction). Types of anesthesia, preoperative antibiotics, surgeons’ years’ experience, PCNL operation details were identified and recorded. Postoperative patients’ data were revised, collected and recorded.

Patients information were collected from our hospital’s database, including age, sex, body mass index (BMI), comorbidities (diabetes, hypertension and coronary heart disease), stone size and laterality, prior indwelling stent, blood tests (cholesterol, creatinine, albumin and globulin levels and white blood cell count), fever (defined as body temperature >38 °C), urine tests (white blood cell and nitrite), urine culture, and ASA score.

Sepsis was defined according to the 2001 International Sepsis Definitions Conference: occurrence of an infection and a minimum of two of the following within 48 h of surgery: (1) heart rate >90/min, (2) body temperature >38 °C, (3) leukocyte count <4,000 cells/μL or >12,000 cells/μL and (4) respiratory rate >20/min (Levy et al., 2003).

All statistical analyses were performed using SPSS 24.0 and R software 3.6.2. The cut-off value was determined by the Youden Index. The student *t*-test was used to detect differences between continuous variables with a normal distribution. The chi-square test or Fisher’s exact test was used to compare categorical variables. The multivariable logistic regression method was used to determine independent risk factors for sepsis. Then predictive nomogram was generated based on converting the regression coefficient to a 0–100-point scale proportionally. The predictive performance of the model was measured by validation, discrimination and decision analysis (Steyerberg & Vergouwe, 2014). A calibration curve was generated with 1,000 bootstrap samples to reduce the overfit bias. Hosmer–Lemeshow (HL) test implied good calibration when the test is insignificant. The discriminative performance was assessed by area under the receiver operating characteristic (ROC) curve. The clinical usefulness of the nomogram is evaluated by decision curve analysis (DCA) by assessing net benefits at different threshold probabilities. *P*-value < 0.05 was considered significant.

## Results

We identified 745 patients who underwent PCNL for a solitary, unilateral, and proximal ureteral stone, and 35 patients developed sepsis. The patients’ demographic details and univariable analysis results of risk factors are shown in Table 1. No patients were concomitant with paraplegia. The characteristics of patients who developed sepsis included female sex, lower albumin, higher globulin, lower AGR (<1.5), preoperative fever, leukocytosis (WBC ≥ 10,000 cells/μL), positive urine culture, leukocyturia (≥50 cells/μL), and positive urine nitrite. Notably, stone size and operation time were comparable between the two groups. The stone shape is rotundity or extension.

**Table 1:** Patients characteristics and univariable analysis of risk factors for post-operative sepsis after percutaneous nephrolithotomy.

Variable	Sepsis (*n* = 35)	Non-sepsis (*n* = 710)	*P* value
Age (year), mean (SD)	49.7 (10.8)	50.7 (12.1)	0.625
BMI (kg/m^2^), mean (SD)	23.8 (3.7)	23.9 (3.0)	0.863
Operation time (min), mean (SD)	107.6 (32.8)	101.6 (37.2)	0.349
Sex, *n* (%)			<0.001
Male	11 (2.3)	462 (97.7)	
Female	24 (8.8)	248 (91.2)	
Stone height, *n* (%)			0.775
<20 mm	29 (4.6)	601 (95.4)	
≥20 mm	6 (5.2)	109 (94.8)	
Stone laterality, *n* (%)			0.911
Left	18 (4.6)	372 (95.4)	
Right	17 (4.8)	338 (95.2)	
Indwelling stent, *n* (%)			0.917
Yes	3 (6.0)	47 (94.0)	
No	32 (4.6)	663 (95.4)	
Hydronephrosis, *n* (%)			0.268
Yes	4 (2.9)	134 (97.1)	
No	31 (5.1)	576 (94.9)	
Hypertension, *n* (%)			0.556
Yes	7 (3.9)	173 (96.1)	
No	28 (5.0)	537 (95.0)	
Coronary heart disease, *n* (%)			1.000
Yes	1 (7.1)	13 (92.9)	
No	34 (4.7)	697 (95.3)	
Diabetes, *n* (%)			1.000
Yes	3 (4.8)	60 (95.2)	
No	32 (4.7)	650 (95.3)	
Serum cholesterol, *n* (%)			0.128
<5.17 mmol/L	34 (5.2)	617 (94.8)	
≥5.17 mmol/L	1 (1.1)	93 (98.9)	
Serum creatinine, *n* (%)			0.541
Normal	31 (4.9)	602 (95.1)	
Abnormal	4 (3.6)	108 (96.4)	
Albumin, *n* (%)			0.005
<35 g/L	10 (11.2)	79 (88.8)	
≥35 g/L	25 (3.8)	631 (96.2)	
Globulin, *n* (%)			0.001
<30 g/L	12 (2.6)	450 (97.4)	
≥30 g/L	23 (8.1)	260 (91.9)	
AGR, *n* (%)			<0.001
<1.5	33 (6.7)	457 (93.3)	
≥1.5	2 (0.8)	253 (99.2)	
Pre-operative fever, *n* (%)			<0.001
Yes	9 (17.0)	44 (83.0)	
No	26 (3.8)	666 (96.2)	
WBC, *n* (%)			<0.001
<10,000 cells/μL	25 (3.7)	659 (96.3)	
≥10,000 cells/μL	10 (16.4)	51 (83.6)	
Urine culture, *n* (%)			<0.001
Positive	13 (14.9)	74 (85.1)	
Negative	22 (3.3)	636 (96.7)	
Urine WBC, *n* (%)			0.001
<50 cells/μL	7 (1.9)	353 (98.1)	
≥50 cells/μL	28 (7.3)	357 (92.7)	
Urine nitrite, *n* (%)			< 0.001
Positive	8 (17.8)	37 (82.2)	
Negative	27 (3.9)	673 (96.1)	
ASA score, *n* (%)			0.591
1	18 (5.9)	286 (94.1)	
2	17 (4.0)	408 (96.0)	
3	0 (0.0)	15 (100.0)	
4	0 (0.0)	1 (100.0)	

**Note:**

BMI, body mass index; SD, standard deviation; AGR, albumin globulin ratio; WBC, white blood cell.

**Figure 1:**
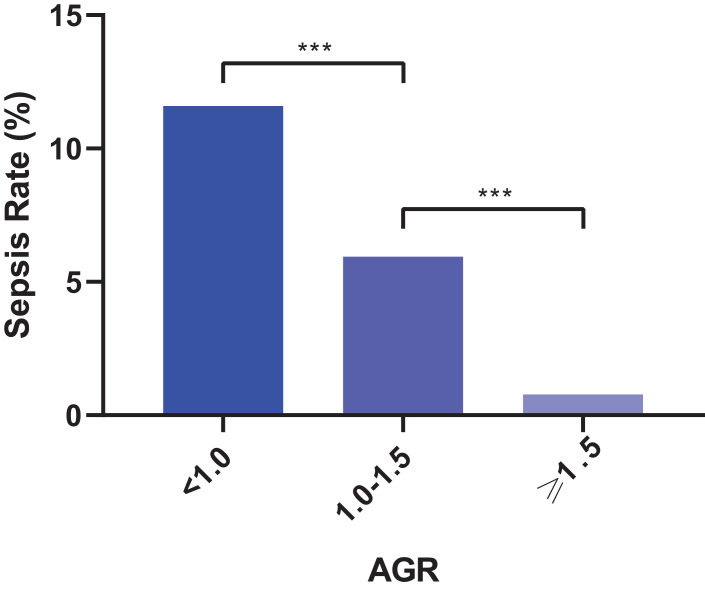
Categorized albumin globulin ratio (AGR) and corresponding sepsis rate (***P < 0.001).

In the multivariate analysis, four factors were identified as independent factors for sepsis: AGR < 1.5 (odds ratio [OR] = 5.068, 95% confidence interval [CI] [1.135–22.624], *P* = 0.033), positive urine culture (OR = 3.243, 95% CI [1.162–9.047], *P* = 0.025), leukocytosis (OR = 3.706, 95% CI [1.444–9.512], *P* = 0.006) and female sex (OR = 2.529, 95% CI [1.127–5.672], *P* = 0.024) (Table 2). Furthermore, we categorized AGR into three groups: ≥1.5, 1.0–1.5 and <1.0. Figure 1 shows an increasing sepsis rate with increasing levels: 0.78% (2/255), 5.93% (25/421) and 11.59% (8/69), respectively.

**Table 2:** Multivariable logistic regression analysis of predictors of sepsis after percutaneous nephrolithotomy.

Variables	B	SE	OR	95% CI	*P* value
Age (year)	−0.021	0.018	0.979	[0.946–1.014]	0.240
BMI (kg/m^2^)	0.021	0.059	1.021	[0.909–1.148]	0.720
Operation time (h)	0.273	0.297	1.314	[0.734–2.351]	0.358
Stone height ≥20 mm	0.034	0.502	1.035	[0.387–2.768]	0.945
Pre-operative fever	0.828	0.476	2.288	[0.900–5.820]	0.082
Indwelling stent	−0.728	0.684	0.483	[0.127–1.844]	0.287
Diabetes	−0.010	0.700	0.990	[0.251–3.906]	0.989
Hydronephrosis	0.214	0.572	1.238	[0.404–3.797]	0.709
AGR<1.5	1.623	0.763	5.068	[1.135–22.624]	0.033
Positive urine culture	1.176	0.523	3.243	[1.162–9.047]	0.025
Leukocytosis	1.310	0.481	3.706	[1.444–9.512]	0.006
Positive urine nitrite	0.052	0.581	1.053	[0.337–3.287]	0.929
Female	0.928	0.412	2.529	[1.127–5.672]	0.024
Urine WBC ≥ 50 cells/μ	0.706	0.481	2.026	[0.789–5.197]	0.142

**Note:**

AGR, albumin globulin ratio; WBC, white blood cell; B, regression coefficient; SE, standard error; OR, Odds Risk; CI, confidence interval.

**Figure 2:**
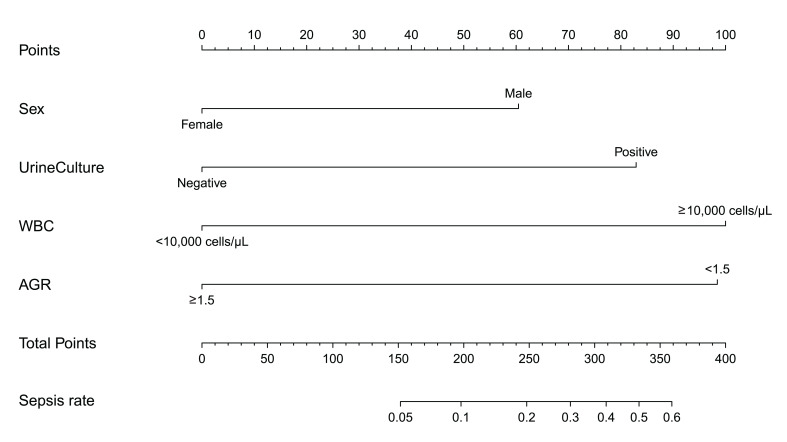
Nomogram for patients predicting post-operative sepsis. WBC, white blood cell; AGR, albumin globulin ratio. Sex, urine culture, WBC and AGR are marked as “points”. Total points by adding the four points can predict sepsis rate.

**Figure 3:**
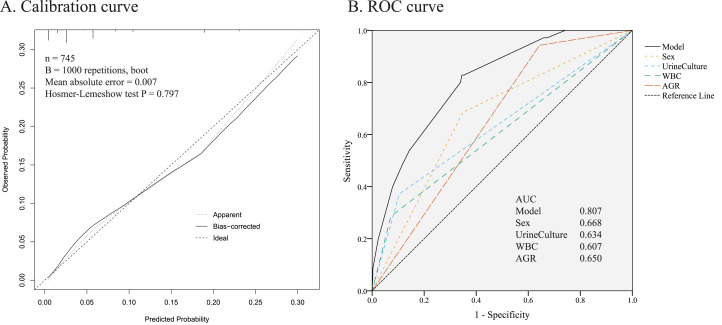
Evaluation of the predictive performance. (A) Calibration curve. Hosmer–Lemeshow test with insignificant *P* value indicates good fitting of model. (B) Receiver operating characteristic (ROC) curve. The area under curve (AUC) for the model is 0.807, which showed a favorable ability of discrimination.

**Figure 4:**
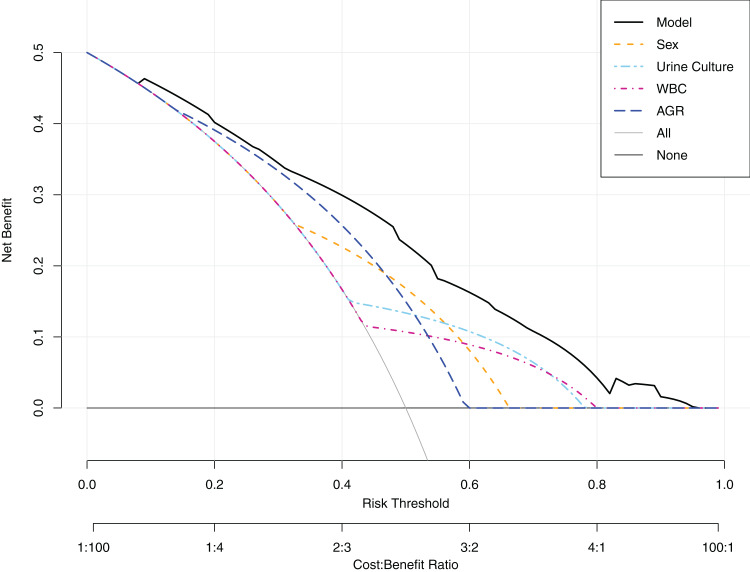
Decision curve analysis (DCA). When risk threshold is around 10–90%, the net benefit of application of the model on taking measures is greater than “treat-all-patient” or “treat-none” scheme. In addition, utilization of sex, urine culture, white blood cell (WBC) and albumin globulin ratio (AGR) alone is inferior than the model.

Based on the multivariable analysis, a nomogram prediction model was established to calculate the cumulative probability of sepsis after PCNL (Fig. 2). The total points were obtained by adding the points assigned to the four factors. The corresponding sepsis rate is indicated by the total points axis. The calibration curve showed good fitting of the model with no statistical significance (*P* = 0.797) through the HL test (Fig. 3A). The ROC curve illustrated favorable discrimination with an area under the curve of 0.807 (Fig. 3B). The DCA of the model showed a threshold probability of 10–90% (Fig. 4), in which the model had the ability to identify stone patients who might develop sepsis after PCNL superior to the “treat-all-patients” or “treat-none” schemes.

## Discussion

Compared with other minimally invasive lithotripsy techniques, PCNL is generally considered to be a safe technique that offers the highest stone-free rates (Wiesenthal et al., 2011; De et al., 2015). However, complications still occur following this percutaneous procedure. With the advancements in technology in recent years, the safety of PCNL has been effectively improved. However, infection is still one of the main complications. The sepsis rate reached 4.7% (35/745) in our research, which was consistent with previous studies (0.9–5.9%) (Rivera et al., 2016; Shoshany et al., 2015). Once the infection further deteriorates without timely recognition and intervention, it may lead to life-threatening complications, like shock and organ dysfunction. Therefore, the early determination of factors associated with sepsis complications is critical to avoid serious postoperative events. In this study, we developed a nomogram utilizing the four preoperative characteristics from multivariate analysis: sex, AGR, urine culture and blood WBC. This preoperative predictive model combined both local and systematic conditions to evaluate the probability of sepsis.

Interestingly, our study revealed that AGR (<1.5) is an independent predictor of post-PCNL sepsis (RR = 5.068, *P* = 0.033). A low AGR is mainly used as a predictor of cancer progression and cancer-related mortality (Lv et al., 2018). AGR mainly reflects the changes in plasma albumin and globulin levels. Among them, albumin is used to reflect the nutritional status and systemic inflammatory response; a lower level of albumin leads to the insufficient synthesis of immunoglobulin, which weakens the immune system (McMillan et al., 2001). Yang et al. (2017) reported that a low-normal level of serum albumin before surgery predicts post-PCNL fever. Xu et al. (2019) demonstrated that the preoperative high-sensitive C-reactive protein/albumin (hs-CRP/Alb) ratio is independently predictive for the development of systemic inflammatory response syndrome after PCNL. Usually, globulin plays an important role in immunity and inflammation. In the early phase of infection, the immunoglobulin level elevated rapidly (Nevo et al., 2017), which may account for the results in our study. A low AGR may indicate a susceptible state to infection. PCNL surgery brings about a stress response and backflow of pathogens and toxins, which accelerates the spread of infection and further leads to the occurrence of sepsis. Unlike other studies that only focus on local factors (Rashid & Fakhulddin, 2016; Sharma et al., 2016), our study found that AGR, a systemic factor, maybe one of the important predictive factors of sepsis after PCNL.

Another systemic factor noted in our study to be closely related to sepsis after PCNL is preoperative leukocytosis (OR = 3.706, *P* = 0.006). Mahmood et al. (2017) reported that preoperative leukocytosis is associated with adverse postoperative outcomes after cardiac surgery and is an independent predictor of infection-related postoperative complications. Sen et al. (2016) found that the preoperative neutrophil-lymphocyte ratio may be a promising additive predictor of bacteremia and postoperative sepsis in patients who undergo PCNL for renal stones. However, Bozkurt et al. (2015) presented a different view on the relationship between postoperative leukocytosis and sepsis. Their study showed that postoperative leukocytosis is common after PCNL and represents a normal physiologic response to surgery. Preoperative leukocytosis may indicate the existence of potential infection in the body. The stress reaction and trauma caused by PCNL surgery may lead to the aggravation of infection, and thus, resulting in sepsis.

As a usual local urological condition, positive urine culture is considered as a critical factor related to infectious complications after a PCNL operation (Yang et al., 2017; Uchida et al., 2018). It is in conformity with our result that the risk of sepsis was found to be increased by more than three times in patients with positive urine culture. PCNL may cause vascular injury and high intrarenal pressure. For patients with a positive urine culture, this operation can promote the invasion of blood circulation by local pathogens and toxins and lead to the occurrence of sepsis. In our study, bladder urine was used for the urine culture examination. Emerging studies concluded that stone culture (Eswara, Shariftabrizi & Sacco, 2013; Mariappan et al., 2005) and pelvic urine culture (Mariappan et al., 2005) were better predictors of urosepsis than mid-stream bladder urine culture. However, sample from kidney and stone cannot be obtained until surgery starts, which limits their early predictive and preventive value. Mid-stream bladder urine culture is still the optimal predictor that can be early acquired.

In our study, female sex was also found to be an independent predictor for sepsis after PCNL (OR = 2.529, *P* = 0.024). Similarly, many studies have reported that female sex was significantly associated with post-URS or post-PCNL SIRS (Xu et al., 2019; Uchida et al., 2018; Liu et al., 2013). Martov et al. (2015) from the CROES URS Global Study showed that female sex was a significant risk factor of postoperative urinary tract infection or fever in patients with a negative baseline urine culture. Female is more susceptible to urinary tract infection than male, which may be one of the reasons why female sex is prone to sepsis after PCNL (Zhu et al., 2019). Additionally, sex-related gene polymorphisms (Hubacek et al., 2001) and the effects of sex hormones (Federman, 2006) may be other potential pathomechanisms to account for the susceptibility to sepsis after PCNL of the female sex.

Our study had certain limitations. First, this study was a retrospective study performed at a single center, which may have led to potential selection bias. Second, only patients with proximal ureteral stone were enrolled considering homogeneous stone characteristics. Further research will investigate various kidney stone to verify the risk factors of sepsis. Third, although the model established in this study revealed favorable fitting, discrimination and clinical usefulness, external validation is lacking, which is expected to be testified in independent cohorts. It is the first study to reveal the predictive value of the preoperative AGR for post-PCNL sepsis. However, the specific underlying mechanism remains unclear. A prospective study is planning to uncover the septic risk with more precise variables.

## Conclusions

A new nomogram is developed to predict sepsis in PCNL for an upper ureteric single stone if the patient has these characters: female sex, lower AGR, positive urine culture and leukocytosis.

## Supplemental Information

10.7717/peerj.9435/supp-1 Supplemental Information 1 Raw data: all patient information collected from the hospital’s database.

## Additional Information and Declarations

### Data Availability

The following information was supplied regarding data availability: The raw measurements are available as a Supplemental File.
